# Traumatic Tetanus

**Published:** 1885-09

**Authors:** 


### TRAUMATIC TETANUS—RECOVERY.

We see reported in the New York *Medical Record*, by Dr. J. G. Starr, of Illinois, a case of traumatic tetanus successfully treated by bromide potassium, chloral hydrate and fluid extract physostigmus. Morphine was given also, and the wound, after being freely incised, was cauterized with nitrate silver and nitric acid, and poulticed. We are reminded here of the successful treatment of traumatic tetanus by the lamented Hodgens, by large doses of Fowler's solution. It was so successful that it came to be known as Hodgens' treatment.

